# Rosiglitazone reverses high fat diet-induced changes in BMAL1 function in muscle, fat, and liver tissue in mice

**DOI:** 10.1038/s41366-018-0090-5

**Published:** 2018-05-24

**Authors:** Aleix Ribas-Latre, Baharan Fekry, Christopher Kwok, Corrine Baumgartner, Samay Shivshankar, Kai Sun, Zheng Chen, Kristin Eckel-Mahan

**Affiliations:** 1 The Brown Foundation Institute of Molecular Medicine, McGovern Medical School at the University of Texas Health Science Center, Houston, TX 77030 USA; 2 Program of Biochemistry and Cell Biology, The Graduate School of Biomedical Sciences at the University of Texas Health Science Center, Houston, TX 77030 USA; 3 Department of Biochemistry and Molecular Biology, McGovern Medical School at the University of Texas Health Science Center, Houston, TX 77030 USA

**Keywords:** Physiology, Type 2 diabetes, Obesity

## Abstract

**Objective:**

Nutrient challenge in the form of a high fat (HF) diet causes a reversible reprogramming of the hepatic circadian clock. This depends in part on changes in the recruitment of the circadian transcription factor BMAL1 to genome targets, though the causes and extent of disruption to hepatic and extra-hepatic BMAL1 are unknown. The objective of the study was to determine whether HF diet-induced alterations in BMAL1 function occur across insulin-resistant tissues and whether this could be reversed by restoring whole body insulin sensitivity.

**Methods:**

BMAL1 subcellular localization and target recruitment was analyzed in several metabolically active peripheral tissues, including liver, muscle, and adipose tissue under conditions of diet-induced obesity. Animals made obese with HF diet were subsequently treated with rosiglitazone to determine whether resensitizing insulin-resistant tissues to insulin restored hepatic and extra-hepatic BMAL1 function.

**Results:**

These data reveal that both hepatic and extra-hepatic BMAL1 activity are altered under conditions of obesity and insulin resistance. Restoring whole body insulin sensitivity by treatment with the antidiabetic drug rosiglitazone is sufficient to restore changes in HF diet-induced BMAL1 recruitment and activity in several tissues.

**Conclusions:**

This study reveals that a key mechanism by which HF diet interferes with clock function in peripheral tissues is via the development of insulin resistance.

## Introduction

Circadian rhythms are evident in almost every aspect of physiology. Such rhythms, exemplified by the sleep/wake cycle, energy intake, body temperature, etc., are sustained by 24-h rhythms in individual cells of the body. Oscillations within the cell occur at almost every level, including chromosomal dynamics and transcription [1], posttranscriptional and posttranslational processing [2, 3], and nutrient metabolism [4]. Cellular rhythms are driven in large part by a negative transcriptional feedback loop involving the heterodimerization of CLOCK and BMAL1 transcription factors, which together drive 24-h oscillations in target circadian and metabolic genes, including at genomic loci encoding their own negative regulators, the cryptochrome (*Cry*) and period (*Per*) genes [5]. Coordination of rhythms across groups of cells and ultimately across tissues is essential for metabolic health. Epidemiological studies reveal that night shift workers, rotating shift workers, and otherwise “socially jet-lagged” individuals have an increased risk of metabolic disorders, though the complex mechanisms underlying this association are an area of active investigation [6].

While the light dark cycle is the primary entrainment mechanism for the SCN, maintenance of circadian rhythms in several highly metabolic peripheral tissues depends heavily on nutrient intake [7–12]. In fact, nutrients can override the SCN’s directive influence in some peripheral tissues [7, 8, 13], reviewed in refs. [14, 15]. For example, temporal restriction in energy intake can restore 24-h gene expression rhythms in an otherwise arrhythmic liver [7, 16]; and altering nutrient quality, such as administering a high fat (HF) diet, can “reprogram” the circadian clock in the liver, as revealed by a genome-wide restructuring of the transcriptome and metabolome [17]. This reprogramming involves both the circadian induction and chromatin recruitment of PPARγ, as well as changes in BMAL1 recruitment to target genes. This altered BMAL1 recruitment is manifest by a phase advance in the occupancy at some target loci, and a loss of recruitment to others [17]. Whether HF-induced reprogramming is due to specific nutrients in the diet vs. the resulting insulin resistance has not been directly demonstrated, and the extent to which other insulin-resistant tissues are affected is not clear from the standpoint of BMAL1 function. Thus, to date, the extent to which direct vs. indirect mechanisms contribute to circadian reprogramming has not been fully elucidated; however, the circadian transcriptional activator, BMAL1, appears to be particularly sensitive to HF diet, showing aberrant binding at target E box sites throughout the genome.

Prior studies in mice reveal that HF diet-induced changes in BMAL1 recruitment can be reversed by a return to low fat diet feeding [17]; but whether this reversal relies on changes in insulin sensitivity has not been demonstrated. Some evidence to date suggests that HF may affect BMAL1-mediated reprogramming by modifying insulin signaling. For example, the kinases AKT2 and GSK3β have been reported to alter BMAL1 activity [18–21], and small molecule inhibitors of GSK3β induce a phase advance of the clock, as measured by *Per2* promoter-driven gene expression [22], similar to the hepatic phase advance that occurs under HF diet [17, 23]. Similarly, rats treated with streptozotocin, lethal to pancreatic beta cells, show a similar phase advance of circadian genes in the liver, muscle, and adipose tissue, while such phase changes are not mirrored in less insulin responsive tissues, such as the lung [24].

In this study, we tested whether HF diet affects BMAL1 function in an insulin-dependent manner by analyzing BMAL1 activity in the liver as well as in several extra-hepatic tissues under conditions of diet-induced obesity with or without application of the thiazolidinedione, rosiglitazone (ROSI). Our results reveal that HF diet alters BMAL1 recruitment and activity not only in the liver but also in the muscle and white adipose tissue, and that antidiabetic treatment that restores whole body insulin sensitivity is able to reverse the aberrant BMAL1 activity in these tissues.

## Materials and methods

### Animals

Procedures related to the care and use of mice were reviewed and approved by the McGovern Medical School, UT Health Science Center Animal Care and Use Committee, protocol AWC-15-0091. Male, C57BL/6J mice purchased from Jackson (000664) were allowed to eat and drink ad libitum unless otherwise indicated. Animals were maintained on a 12-h light (L)/12-h dark (D) schedule unless otherwise indicated. For all experiments, zeitgeber (ZT) 0 refers to the light onset in the room, corresponding to the animals’ sleep/fasting phase. ZT12 refers to the onset of darkness, the beginning of the animals’ active/feeding phase. Generally, mice were between 6 and 8 months of age at the time of experimentation.

### Experimental designs

#### Experimental design 1

**Fig. 1:**
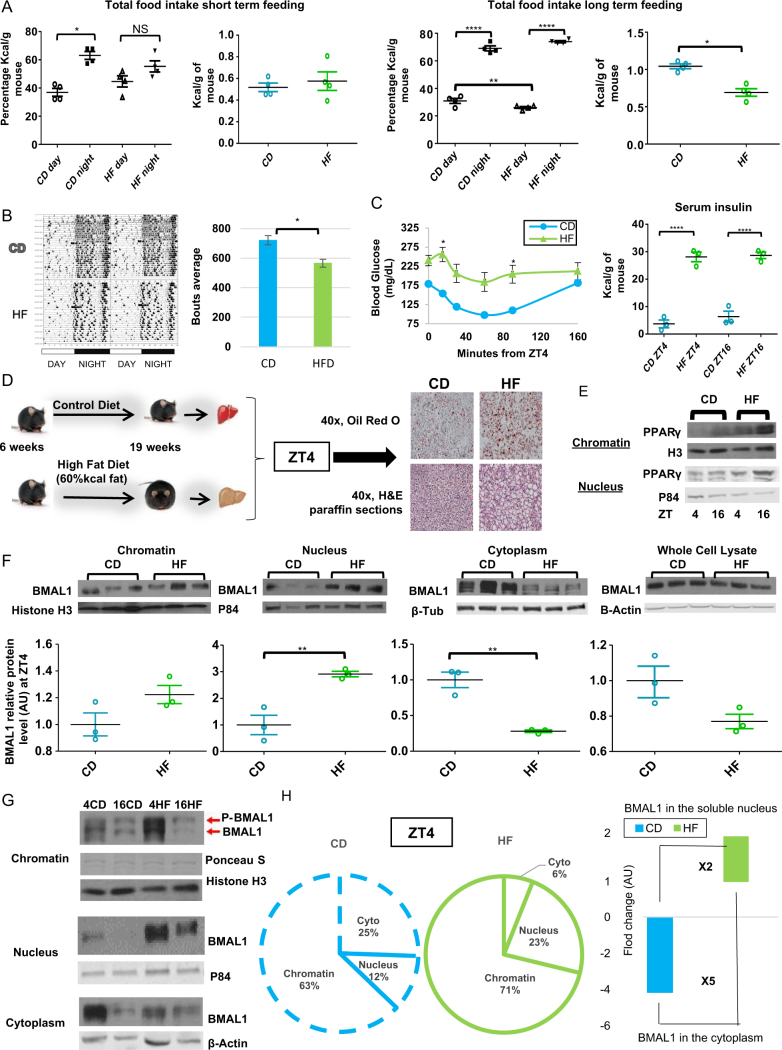
Adaptation to high fat (HF) diet restores circadian energy intake. **a** Quantification of food intake (*n* = 4) after transient (left panels) and chronic (right panels) CD and HF feeding. Significance (*p* < 0.05) was determined by Mann–Whitney *U*-test (transient intake and right panel for chronic intake) and two-way ANOVA followed by Tukey’s post-hoc test (left panel, chronic intake). **b** Actograms (*n* = 8) reveal home cage activity of animals on CD and HF (left panel). Quantification, right panel. Significance (*p* < 0.05) was determined by Mann–Whitney *U*-test. **c** Circulating glucose levels (*n* = 8) measured during an IP insulin tolerance test (left panel). Serum insulin levels of CD (*n* = 3) and HF (*n* = 4) mice, right panel. Significance (*p* < 0.05) was determined by two-way ANOVA followed by Tukey’s post-hoc test. **d** Model of diet-induced obesity (DIO) (left panel) and Oil Red O and H&E staining from DIO mouse livers (right panel) at ZT4. **e** Western blot reveals diurnal expression of Pparγ in the chromatin and soluble nuclear compartments of CD and HF animals. **f** Western blot reveals compartmentalization of hepatic BMAL1, as well as levels in whole cell lysates of individual animals (*n* = 3) at ZT4 undergoing prolonged HF feeding compared to CD. Western blot quantification in arbitrary units (AU), lower panels. Significance (*p* < 0.05) was determined by unpaired Student’s *t*-test. **f** Western blotting reveals compartmentalization of BMAL1 in the liver of animals at ZT4 and ZT16 after chronic CD and HF. **g** Pie charts, normalized by the total BMAL1 levels in the whole cell lysates, express changes in BMAL1 subcellular localization after chronic CD vs. HF feeding (left panel). Representation of the fold change in cytoplasmic and nuclear BMAL1 under CD and HF feeding (right panel). *p < 0.05, **p < 0.01, ***p < 0.001, ****p < 0.0001

Fig. 1, S1, and 2: male mice were exposed to control diet (CD, Research Diets D12450B, NJ, US) or HF (Research Diets D12492, NJ, US) starting at 8 weeks of age. Glucose tolerance tests (GTT) and insulin tolerance tests (ITT) were administered pre- and post-diet change. Animals were fed the corresponding diets for 13 weeks, during which time a separate cohort of animals were tested for metabolic activity as described in “metabolic phenotyping.” Mice were killed after 13 weeks on the diet at ZT4 and ZT16. At killing, trunk blood was collected and allowed to clot. Blood was spun for 30 min at 4000 r.p.m. and serum was collected and stored at −80 °C for subsequent analysis. Livers and subcutaneous fat pads were removed and immediately placed in liquid nitrogen, prior to permanent storage at −80 °C for further analysis. (Separate sets of animals were analyzed by infrared sensors (Fig. 1b) or wheel runners (Figure S1D), to study diurnal and circadian activity on each diet, as described in the “Circadian activity” section.)

#### Experimental design 2

Fig. 3, S2 and 4: male mice on vivarium chow (VC, Pico Lab rodent diet 20, 5058, MO, US) or HF diet (Research Diets D12492, NJ, US) for 13 weeks were treated with vehicle or ROSI (10 mg/kg body weight, Cayman Chemical, 71740, MI, US) for 2 weeks. All injections were performed at ZT0. Animals were administered GTT and ITT before and after administration of ROSI to test for glucose and insulin tolerance. Animals were killed at ZT4. Livers, gastrocnemius muscle, hippocampus, and the visceral and subcutaneous fat pads were removed and immediately placed in liquid nitrogen, and subsequently stored at −80 °C for further analysis.

#### Insulin injection experiment

Lean, male animals fed with VC were fasted for 4- h and then injected intraperitoneally with insulin (0.75 U/kg) or vehicle at ZT4. Mice were killed at the indicated time points following injection and livers were flash frozen in liquid nitrogen. Livers were stored at −80 °C and subsequently prepared for cytoplasmic and nuclear fractionation.

### Circadian activity

Mice were singly housed in cages containing 4″ diameter wheel runners or infrared sensors (STARR Life Sciences, PA) to monitor circadian wheel running or total activity, respectively. While experiments done only in 12-h L/12-h D were performed in cages equipped with infrared sensors (to assess the relative level of total home cage activity during the rest period), locomotion in free running conditions was analyzed using cages equipped with wheel runners, to better assess potential changes in period length (See supplementary S1). Data were collected using VitalView (STARR Life Sciences) and locomotion data were analyzed using ClockLab software (ActiMetrics, IL).

### Feeding regimens

Mice were fed one of three diets: VC (Pico Lab rodent diet 20, 5058, MO, US), CD (Research Diets D12450B, NJ, US), and HF (Research Diets D12492, NJ, US) diets. Independent experiments were performed to confirm that 13 week exposure to VC and CD resulted in the same degree of whole body insulin sensitivity.

### Metabolic phenotyping

Mice were placed at room temperature (22–24 °C) in metabolic cages (Comprehensive Lab Animal Monitoring System-CLAMS; Columbus Instruments) and VC was replaced with ground versions of diets immediately upon recording for short-term feeding analysis. For long-term feeding analysis, metabolic phenotyping was performed 6 weeks after the diet switch. Food and water were provided ad libitum. Food intake and energy expenditure data were collected and averaged over the course of 4 days. Animals maintained on their respective diets were placed back into the chamber five weeks later, and energy expenditure and food and water intake were again monitored. Data were analyzed using Oxymax V 4.87.

### Glucose and ITT

For the GTT, mice were fasted for 5-h and injected intraperitoneally with glucose (1 g/kg). Blood from the tail was collected and measured at time points indicated using a glucose meter (ACCU-CHEK Nano). For the ITT, mice were fasted for 4-h and then injected intraperitoneally with insulin (0.75 U/kg). Glucose levels were measured at indicated time points following injection. Access to food was denied during the course of the studies. All GTT and ITT procedures were performed between ZT7-9.

### Insulin quantification

Insulin levels in the serum were quantified using an ELISA kit following the manufacturer’s instructions (Millipore, MA, US).

### Oil Red staining

Frozen liver sections (5 µM) were fixed in 10% formalin for 60 min at room temperature and rinsed three times with phosphate-buffered saline (PBS) for 5 min each. Slides were incubated in ORO liquid for 15 min followed by three rinses in PBS for 5 min each. Slides were dipped in 75% alcohol for 2 s, and then washed with water for 1 min. Cell nuclei were stained with Harris hematoxylin for 2 min and then washed with tap water. Slides were placed in 1% hydrochloric-alcohol solution for several seconds and washed with tap water several times until the water ran clear. Slides were cover-slipped and sealed.

### Histology

Liver sections (5 µM) were fixed in formalin and embedded in paraffin. Liver sections were washed twice with xylene for 3 min and rehydrated in ethanol gradients (100%, 2 × 5 min; 90%, 1 × 5 min; 80%, 1 × 5 min; and 70%, 1 × 5 min). Sections were subsequently stained with hematoxylin and counterstained with eosin-phloxine solution. All images were acquired with an Axio Scope A1 microscope using ZEISS microscope software ZEN2 (Carl Zeiss, Oberkochen, Germany).

### Protein extraction and fractionation

For whole cell lysates, liver tissue was homogenized in RIPA lysis buffer (see Supplementary Materials) for 15 s using a MagNA Lyser (Roche, IN, US). Samples were nutated for 15 min and spun at high speed for 15 min to eliminate insoluble material. For tissue fractionations, liver tissue was homogenized for 15 s in buffer A (BA, see Supplementary Materials) using a MagNA Lyser. Samples were spun at low speed for 10 min and supernatant was saved for cytoplasmic fractions. Pellets were resuspended in 1 ml of BA and spun at low speed for 20 min. Pellets were washed 2× with low salt buffer (LSB) and the final pellet was dissolved in 150 µl LSB per 100 µl pellet. The resulting homogenate was resuspended vigorously in 2× volume high salt buffer (HSB) and nutated for 1 h at 4 °C. Samples were spun for 20 min at 10,000 × *g* and the soluble nuclear material (supernatant) was snap frozen in liquid nitrogen and stored at −80 °C. Remaining pellets were resuspended in RIPA lysis buffer and sonicated (Qsonica, Newton, US) for 10 s, amplitude 30%. Samples were spun at 10,000 × *g* for 5 min to remove insoluble material and the resulting chromatin material was stored at −80 °C (Note: LSB and HSB components can be found in supplementary materials and methods.).

### Western blotting

Protein concentration was determined using BCA (Thermo Scientific IL, US). Lysates were diluted with RIPA (total protein and chromatin fractions), BA (cytoplasm), or Low-HSB (nuclear fraction) and 5X Laemmli (0.5 M Tris-HCl, pH 6.8; 10% glycerol; 2% (w/v) SDS; 5% (v/v) β-mercaptoethanol; and 0.05% bromophenol blue). Generally, 25 μg of total and cytoplasm protein, 15 µg of chromatin, and 6 µg of nuclear protein were loaded and separated on a 8% SDS-polyacrylamide gel and transferred to nitrocellulose (Bio-Rad, 162-0115, CA, US). For further western blotting procedures, including antibodies, see Supplemental Materials and Methods, Table S1, and Table S2.

### RNA extraction and reverse transcriptase, quantitative PCR

Total RNA from tissues was isolated as previously described [25]. cDNA was synthesized using iScript (Bio-Rad, CA, US), and product was subjected to quantitative PCR amplification using SsoAdvanced Universal SYBR Green Supermix Mix (Bio-Rad, CA, US), according to manufacturer’s suggestions. The eukaryotic ribosomal subunit 18S was used as an internal control. Data were converted and normalized to the linear form by the 2^−−^CT (∆∆CT) calculation. For additional details including primer sequences, see Supplementary Material and Methods and Table S3.

### Chromatin immunoprecipitation (ChIP)

ChIP from liver was performed as described in ref. [26]. For adipose tissue ChIP, 200 mg of starting material was used and sonication was performed as specified in ref. [27].

### Chemicals

ROSI (Cayman Chemical, MI, US) was initially diluted in DMSO. Due to its poor solubility, ROSI was then diluted 1:3 with PBS immediately prior to injections to achieve a dose of 0.5 mg/ml. ROSI was made fresh daily and mice were injected daily by IP at ZT0, using a dose of 10 mg/kg.

### Statistical analysis

Data are shown as mean with SEM. Depending on the design, significance (*p* < 0.05) was determined by unpaired Student’s *t*-test (two-tailed) or by the nonparametric Mann–Whitney *U*-test. For experiments involving more than one variable, significance was assessed using two-way ANOVA followed by Tukey’s post-hoc test, as indicated in the figure legends. Data were analyzed using GraphPad Prism 7. Data points that exceeded a predetermined 1.5 standard deviation from the mean were considered outliers. For circadian locomotion and glucose and ITT, sample size was chosen based on the expected variance observed in prior studies. Generally, random assignment was used in animal experiments except when placing them into initial diet groups, where non-randomization was chosen to ensure that each group was weight-matched prior to a switch to HF diet. While some experiments were performed blind to the experimental conditions (i.e., histology), data analysis were generally done by a researcher that was not blind to the condition.

## Results

### HF uniquely alters the hepatic clock, despite rhythmic energy intake

To determine whether altered BMAL1 function under nutrient challenge conditions is affected across insulin-sensitive metabolic tissues, mice were fed with CD or HF diet for over 10 weeks to induce obesity. Based on specific dietary regimens used in previous studies, where HF diet conditions have sometimes been compared to VCs, circadian, and metabolic phenotypes were compared between CD (10% kcal from fat) and HF (60% kcal from fat) to assess rhythmicity in energy intake and metabolism. Importantly, some studies suggest that HF diet, when administered ad libitum, leads to arrhythmic energy intake. Thus, to determine whether circadian food intake differed between CD and HF diet under ad libitum conditions, animals were placed in metabolic cages and monitored after short- and long-term exposure to HF diet. Short-term (i.e., 5 day) feeding experiments revealed that in spite of similar total energy intake in the feeding groups, HF feeding resulted in a dampened diurnal amplitude in energy intake compared to CD (Fig. 1a and S1A). This is consistent with prior reports of dampened circadian rhythms in food intake under ad libitum HF feeding [9]. However, prolonged HF feeding for 6 weeks resulted in a diurnal adaptation, and robust rhythmicity was observed under both CD and HF feeding conditions (Fig. 1a, right panels, and S1A). Thus, while short-term HF diet exposure dampens the diurnal fold change in energy intake, long-term adaptation to the diet is sufficient to reverse this effect. Animals on HF gained weight during the first 10 weeks of feeding, reaching a plateau between 10 and 13 weeks (Figure S1B). Daily rhythms in VO_2_ and VCO_2_ were evident under both feeding regimens, while HF mice showed a low and flat respiratory exchange ratio, indicative of a non-oscillating preference for lipids as the fuel source (Figure S1C). HF mice gained weight throughout feeding, amassing 15 additional grams of body weight compared to CD mice (Figure S1B). Twenty-four hour, home cage locomotion data acquired using infrared sensors and under 12-h L/12-h D conditions, revealed hypolocomotion for HF diet mice (Fig. 1b). A similar trend was observed under “free running” (constant dark) conditions (Figure S1D) when mice were analyzed for wheel running activity. Previous reports reveal that HF diet can induce a period lengthening in mice [28]. While HF diet fed mice showed a trend for longer period length, this did not reach significance (Figure S1E). As expected, long-term HF feeding resulted in impaired glucose tolerance (Figure S1F), impaired insulin tolerance (Fig. 1c), and an elevated and diurnally invariant serum insulin profile (Fig. 1c, right panel). In addition, basal non-stimulated phosphorylation of AKT was elevated under conditions of HF as were total and phosphorylated IRS-1 (Tyr608) (Figure S1G).

Abnormal hepatic lipid deposition is thought to be a key component of insulin resistance in the liver [29]. In addition, diet-induced circadian reprogramming in the liver is thought to be due, in part, to de novo rhythmicity of PPARγ and widespread activation of many PPARγ genomic targets [17]. To compare the effect of CD and HF diets on lipid deposition and diurnal PPARγ activation, CD and HF livers were analyzed for lipid storage. ORO and H&E staining revealed an increase in hepatic lipids under HF diet compared to CD at both fasting and feeding phases examined (Fig. 1d and S1H). In addition, PPARγ target genes involved in lipid storage, such as *Cidec*, were substantially elevated under HF conditions (Figure S1I). HF diet caused an increase in hepatic diurnal PPARγ expression over CD in the chromatin and nuclear compartments, as well as at the level of gene expression (Fig. 1e and S1K). Thus, compared to CD, prolonged HF feeding uniquely induces the diurnal “reprogramming” factors (i.e., expression of PPARγ), in the presence of rhythmic energy intake.

### HF alters hepatic BMAL1 subcellular localization and chromatin recruitment

Because HF diet has been shown to induce premature chromatin targeting of BMAL1 at some genomic loci compared to control feeding conditions [17, 30, 31], livers were collected at ZT4 from CD and HF mice and fractionated into cytoplasmic, soluble nuclear, and chromatin fractions. ZT4 captures this early recruitment of BMAL1 under HF feeding conditions, and thus was a targeted ZT for subsequent experiments. Fractionation experiments revealed a pronounced shift in BMAL1 protein from the cytoplasm into the soluble nuclear compartment at ZT4 (Fig. 1f, g), resulting in an almost 5-fold reduction in cytoplasmic BMAL1 under HF compared to CD (Fig. 1h). Similar to the report by He et al. [32], total cellular BMAL1 did not increase after prolonged HF feeding (Fig. 1f), possibly due to the loss of cytoplasmic BMAL1.

**Fig. 2:**
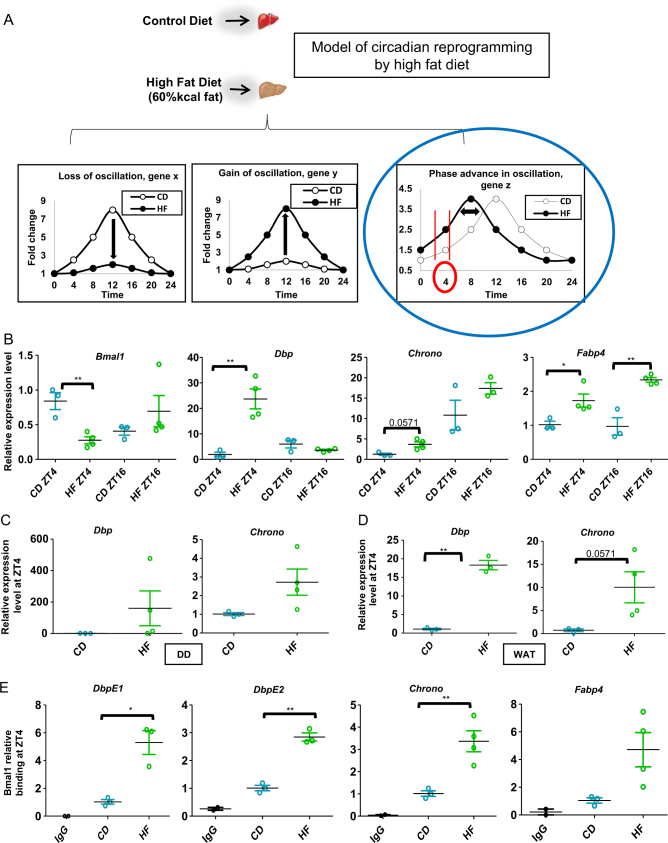
Chronic HF feeding changes BMAL1 subcellular localization and target recruitment during the inactive/light phase. **a** Summary model of circadian reprogramming by high fat diet, includes a phase advance at many BMAL1 targets (circled). **b** qPCR analysis reveals relative mRNA abundance of *Bmal1*, and its targets *Dbp*, *Chrono*, and *Fabp2* in the liver at ZT4 and ZT16 under entrained LD conditions (*n* = 3 for CD and *n* = 4 for HF). Significance (*p* < 0.05) was determined by two-way ANOVA followed by Tukey’s post-hoc test. *Bmal1* and *Chrono* expression at ZT4 were analyzed by an unpaired Student’s *t*-test. **c** qPCR analysis reveals relative mRNA abundance of *Dbp* and *Chrono* in the liver at ZT4 under free running DD conditions (*n* = 4). **d** qPCR reveals relative mRNA abundance of BMAL1 target genes in subcutaneous adipose tissue (SC) of mice on CD (*n* = 3) or HF (*n* = 4) at ZT4. **e** Chromatin immunoprecipitation following by qPCR reveals BMAL1 recruitment at target genes *Dbp*, *Chrono*, and *Fabp2* at ZT4 in the liver (*n* = 3 for CD and *n* = 4 for HF). Significance (*p* < 0.05) was determined by unpaired Student’s *t*-test (**c**–**e**) (*p < 0.05, **p < 0.01, ***p < 0.001, ****p < 0.0001). Relative expression of BMAL1 target genes in CD was set to 1

HF diet-induced changes in the hepatic circadian transcriptome and metabolome involves three primary oscillation categories: a loss of oscillatory gene expression for some genomic loci, a gain of oscillatory gene expression at other loci, and a phase change for many of the remaining oscillatory genes [17] (Fig. 2a). Specifically, the vast majority of this latter category, many of which are known BMAL1 target genes, become phase advanced [17, 23]. Based on this phase advance of the liver clock and the observed changes in subcellular localization of BMAL1 under HF, we sought to determine whether changes in BMAL1 recruitment to specific target sites were induced by HF feeding at ZT4. While HF did not significantly change the overall abundance of chromatin localized BMAL1 (Fig. 1f, g), BMAL1 target genes, *Dbp*, *Chrono (Gm129)*, and *Fabp2* were induced under HF feeding at ZT4 (Fig. 2b), and *Bmal1* mRNA itself, which reaches its nadir during the rest phase, was depressed at ZT4 but elevated at ZT16. To confirm that diurnal changes in gene expression persisted under HF diet in free running conditions, a separate group of animals was maintained in free running conditions and livers were collected at circadian time 4 (CT4) (Fig. 2c). Similar to entrained conditions, BMAL1 target genes were elevated at CT4 under free running (constant dark) conditions. Upregulation of BMAL1 target gene expression was also induced by HF feeding in other insulin-sensitive tissues, including subcutaneous white adipose tissue, where *Dbp* and *Chrono* showed a similar increase in gene expression at ZT4 (Fig. 2d).

To confirm whether increased hepatic BMAL1 target gene expression at ZT4 is due to the early recruitment of BMAL1, we performed ChIP from the liver at ZT4, followed by qPCR using primers flanking known BMAL1 target sites [30]. ChIP revealed an increased recruitment of BMAL1 to target E boxes at ZT4 at *Dbp*, *Chrono*, and *Fabp2* under HF feeding (Fig. 2e). Thus, HF diet induces early recruitment of BMAL1 to some of its chromatin targets and restricts BMAL1 primarily to the nuclear and chromatin compartments at this ZT.

### Reversal of HF-induced changes in hepatic BMAL1 function by ROSI

**Fig. 3:**
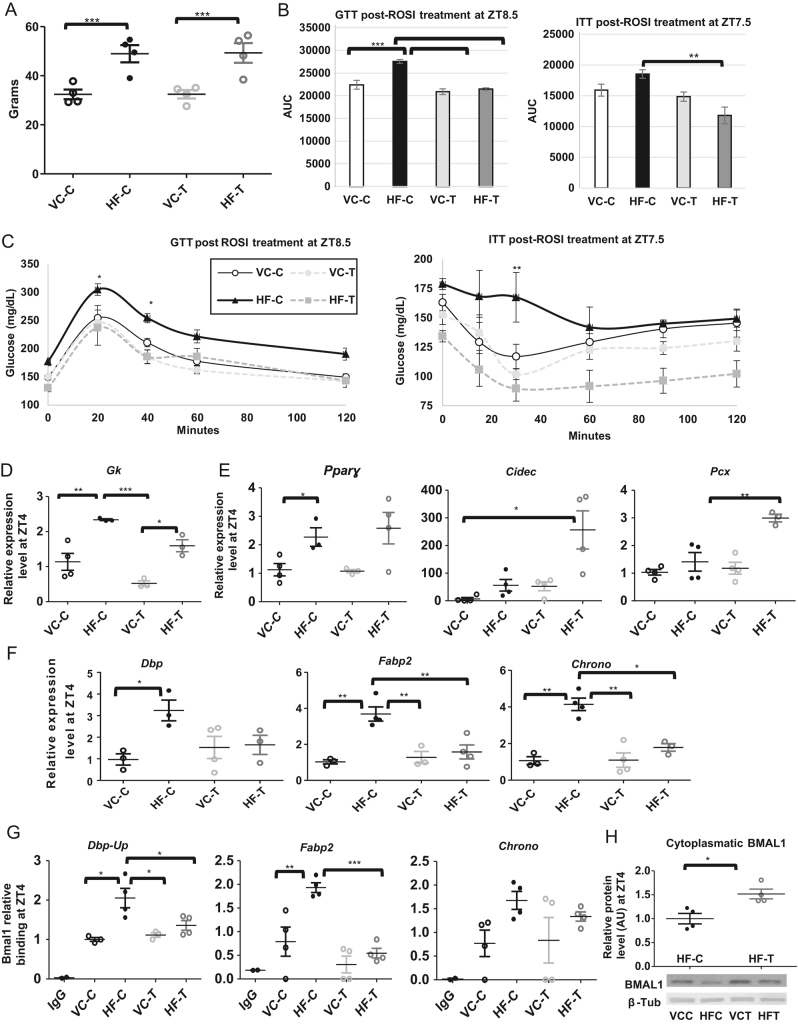
Thiazolidinedione treatment reverses HF-induced alterations in BMAL1 recruitment. **a** Body weight for VC or HF-fed animals administered daily injections of vehicle (C) or ROSI (T) for two weeks (*n* = 4 per condition). Significance (*p* < 0.05) was determined by Mann–Whitney *U*-test. **b** Representation of area under the curve (AUC) from **c** circulating glucose during GTT (left panel) and IPTT (right panel) tests after daily injections of ROSI (T) or vehicle (C) (*n* = 4 per condition). **d** qPCR analysis reveals relative mRNA abundance of *glucokinase* (*Gk*) in the liver of animals treated with ROSI (T) compared to vehicle (C) (*n* = 4 per condition). *Gk* mRNA levels for VC-C were set to 1. **e** qPCR analysis reveals mRNA abundance of *Ppar*γ and its target genes *Cidec* and *Pcx* following vehicle (C) or ROSI (T) treatment (*n* = 4 per condition). Gene expression for *Pparγ* target genes in VC-C were set to 1. **f** qPCR analysis reveals mRNA abundance of *Bmal1* and target genes *Dbp*, *Fabp2*, and *Chrono* following vehicle (C) or ROSI (T) treatment (*n* = 4 per condition). Gene expression for *Bmal1* and target genes in VC-C was set to 1. **g** Chromatin immunoprecipitation of BMAL1 in liver lysates reveals its binding to target E boxes in the *Dbp*, *Fabp2*, and *Chrono* genes after ROSI (T) or vehicle treatment (C) (*n* = 4 per condition). Expression of BMAL1 target genes in VC-C were set to 1. Significance (*p* < 0.05) was determined by two-way ANOVA followed by Tukey’s post-hoc test (**b**–**g**). **h** Western blot reveals BMAL1 protein in the cytoplasm of VC or HF fed mice (*n* = 4 per condition) after treatment with ROSI (T) or vehicle (C) (bottom panel). Quantification in arbitrary units (AU), top panel. Significance (*p* < 0.05) was determined by Mann–Whitney *U*-test comparing HF-C vs HF-T. Relative BMAL1 protein level was set to 1 for HF-C. *p < 0.05, **p < 0.01, ***p < 0.001, ****p < 0.0001

While ROSI has been shown to alter circadian hepatic gene expression [33], we wished to determine whether restoring insulin sensitivity in HF-fed mice is sufficient to restore BMAL1 target recruitment. Chow fed (VC) and obese, HF animals were treated daily with vehicle or the thiazolidinedione, ROSI for 2 weeks. HF feeding induced a gain in body weight (Fig. 3a), which was maintained in both control and ROSI-treated mice. IPGTT and IPITT results revealed impaired glucose and insulin tolerance in vehicle-treated mice on HF diet (Figs. S2A and 3b–c), which was reversed by the 2-week ROSI treatment (expressed as area under the curve (AUC), Fig. 3b). ROSI treatment was also sufficient to reduce *glucokinase* expression in liver, muscle, and subcutaneous WAT (Fig. 3d and S2B). To further determine whether ROSI treatment had direct effects on the liver, we analyzed the expression of *Ppar*γ and some of its direct target genes, *Cidec* and *Pcx*. *Cidec* and *Pcx* expression was induced by ROSI in chow-fed and HF mice (Fig. 3e), most notably under HF diet due to the increase in diurnal PPARγ expression (Fig. 1e and S1J). Thus, ROSI reversed whole body insulin resistance and had direct, local effects on PPARγ in the liver.

To determine whether restoring insulin sensitivity by ROSI was sufficient to restore BMAL1 target recruitment in the livers of HF-fed mice, the BMAL1 target genes, *Dbp*, *Fabp2*, and *Chrono* were analyzed for changes in expression. Strikingly, the elevation in expression of these target genes under HF was almost completely reversed by ROSI treatment, with levels reverting to the chow fed, vehicle-injected mice (Fig. 3f). To determine whether altered gene expression was a result of changes in BMAL1 recruitment, ChIP was performed on livers of chow and HF-fed mice treated with vehicle or ROSI. The early recruitment of BMAL1 to target E boxes was reversed by ROSI (Fig. 3g and S2C), indicating that changes in gene expression after insulin sensitization were a result of altered BMAL1 occupancy at target chromatin. To determine whether ROSI was sufficient to reverse the HF-induced alterations in BMAL1 compartmentalization, livers from vehicle and ROSI-treated animals were fractionated into cytoplasmic and nuclear compartments. This fractionation revealed a ROSI-induced restoration of cytoplasmic BMAL1 under HF at ZT4, concomitant with the reversal of target gene expression (Fig. 3h and S2D). Finally, to confirm that under insulin-sensitive chow-fed conditions insulin is able to alter BMAL1 compartmentalization at this particular ZT window, mice were injected with insulin at ZT4 and livers were collected at 15 and 30 min following insulin injection. Fractionation of the livers from insulin-injected mice revealed a pronounced shift of BMAL1 from the nucleus to the cytoplasm, which was reversed by 30 min after the injection (S2E). Thus, systemic insulin sensitization can alter BMAL1 subcellular localization and chromatin targeting in the liver of animals made obese and insulin resistant by HF feeding.

### ROSI reverses HF diet-induced changes in BMAL1 recruitment in muscle and adipose tissues

**Fig. 4:**
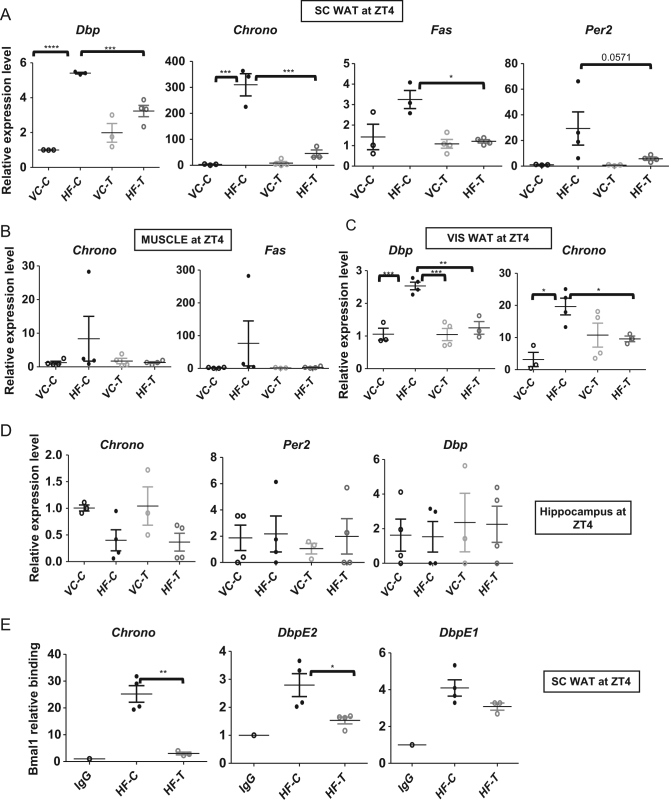
Thiazolidinedione treatment reverses HF diet-induced BMAL1 activity in adipose tissues and muscle. **a**–**d** qPCR analysis of BMAL1 target genes at ZT4 in subcutaneous white adipose tissue (SC WAT, **a**), muscle (**b**), visceral white adipose tissue (**c**), and hippocampus (**d**) in VC and HF-fed mice after daily administration of vehicle (C) or ROSI (T) (*n* = 4 per condition). Significance (*p* < 0.05) was determined by two-way ANOVA followed by Tukey’s post-hoc test with the exception of *Chrono* expression in **c**, where comparison of HF to HFT was analyzed by unpaired Student’s *t*-test. *Bmal1* target gene expression levels for VC-C were set to 1. **e** Chromatin immunoprecipitation followed by qPCR reveals BMAL1 targeting to target E boxes in SC WAT of mice on VC or HF after vehicle (C) or ROSI (T) treatment (*n* = 4 per condition). Significance (*p* < 0.05) was determined by unpaired Student’s *t*-test. IgG is set to 1. *p < 0.05, **p < 0.01, ***p < 0.001, ****p < 0.0001

To determine whether HF induces similar alterations in BMAL1 target gene expression and protein targeting in the muscle and fat tissue, several BMAL1 targets in gastrocnemius muscle and white adipose tissue (subcutaneous and visceral) were analyzed in ROSI and vehicle-treated animals under chow diet and HF. All highly insulin-sensitive tissues examined revealed an increase in the expression levels of these target genes at ZT4, raising the question as to whether similar clock responses to HF diet take place in less metabolically active and insulin-sensitive tissues (Fig. 4a–c). ROSI treatment affected BMAL1 targets similarly to liver in the adipose tissues examined, as well as muscle, reversing the HF-induced increase in BMAL1 target gene expression at ZT4 in these tissues (Fig. 4a–c). Based on the common attribute of these tissues being highly insulin-sensitive and metabolically active, we considered that a less insulin responsive tissue may not show diet-induced alterations in BMAL1 activity. Since similar BMAL1 gene targets are expressed in the brain, including *Per2* and *Chrono*, both of which contribute to circadian rhythms at the level of the central pacemaker [34, 35], we used the hippocampus as a tissue of the central nervous system (CNS) less critical for postprandial glucose uptake to determine whether changes in whole body insulin sensitivity affected the clock at the level of BMAL1 activity. Based on the anatomical position of the SCN and the substantial glucose sensing of the hypothalamic structure as a whole, we chose a CNS region that is not known to have such feeding-dependent glucose sensitivity. Hippocampal lobes from ROSI-treated VC and HF diet mice were analyzed for *Chrono*, *Per2*, and *Dbp* expression. While hippocampal *Chrono* mRNA abundance showed a general trend of less expression under HF, ROSI had no effect at any of the BMAL1 target genes examined (Fig. 4d), further indicating that insulin-sensitive tissues are more subject to diet-induced alterations in clock function at the level of BMAL1 activity.

To verify that ROSI was sufficient to alter BMAL1 occupancy in adipose tissue, ChIP was performed on SC WAT from HF diet fed animals treated with ROSI or vehicle. As for hepatic BMAL1 recruitment, administration of HF diet with ROSI reversed the HF diet-induced BMAL1 binding to target E boxes in SC WAT (Fig. 4e), suggesting that a key mechanism by which diet controls peripheral BMAL1 function similarly in adipose tissue, muscle, and liver is through feeding-induced changes in insulin sensitivity.

## Discussion

**Fig. 5:**
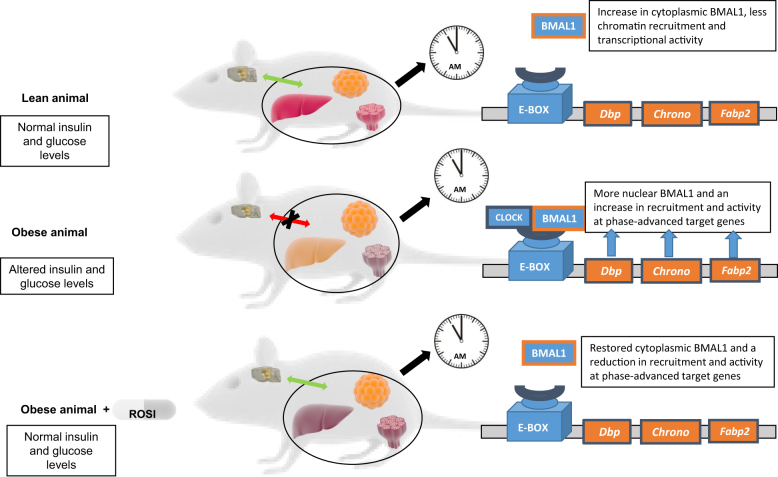
Model of diet-induced changes in BMAL1 activity. During the early resting phase, lean and insulin-sensitive animals under normal chow conditions are limited in BMAL1 chromatin occupancy and transcriptional activation. Under high fat diet feeding, insulin resistance coincides with an early recruitment of BMAL1 to target genes in metabolically active tissues, including the liver, muscle, and adipose tissue. Restoring whole body insulin sensitivity with rosiglitazone is sufficient to reverse the early BMAL1 recruitment and target gene expression patterns in the muscle, adipose tissue, and liver under HF, while having no effect on BMAL1 activity in less insulin-sensitive tissues.

While numerous studies reveal the strong effect of diet on the circadian clock [11, 12, 17, 28, 36, 37], the mechanisms underlying its ability to control circadian rhythmicity has not been entirely clear. Our study adds to several other pieces of evidence suggesting that a tissue’s ability to respond to insulin may be a key determinant of how the clock responds to HF diet. Similar to the effects of HF diet on the clock, small molecule inhibitors of GSK3β have been reported to advance the phase of *Per2* oscillations [22] and STZ-treated rats show a similar phase advance of the clock in liver, muscle, and adipose tissue, but not in less insulin-sensitive tissues [24]. Several kinases within the insulin signaling pathway can affect BMAL1 function [18, 19, 38, 39], but their direct effects at the level of BMAL1 binding across tissues have not been directly assessed. Based on existing data, this study addresses the hypothesis that HF not only affects BMAL1 activity in the liver, but other insulin-sensitive tissues as well, and that aberrant BMAL1 recruitment can be restored in insulin-resistant tissues by restoring whole body insulin sensitivity (Fig. 5). Indeed, HF-induced changes in BMAL1 recruitment occurred similarly across several insulin-resistant tissues, whereas some CNS tissues, such as the hippocampus, were immune to these dietary manipulations.

Inducible loss of BMAL1 in the liver, but also other models of peripheral arrhythmicity reveal that even under entrained (i.e., 12-h L/12-h D) conditions and standard-diet feeding, excess hepatic lipid deposition occurs [40]. In fact, a similar lipid deposition occurs in WT animals undergoing chronic lighting paradigms that mimic jet lag in humans [40]. Thus, direct and indirect regulation of the hepatic clock appears to be important for preventing hepatosteatosis.

A prevailing theory regarding the association of circadian disruption with metabolic disease is that metabolic imbalance is caused not by lack of rhythmicity altogether, but rather loss of circadian coordination across tissues [41]. For example, HF diet-induced reprogramming in the liver involves many de novo oscillations in gene expression and metabolite abundance and a phase advance of other rhythms, but HF feeding results in a profound loss of oscillations for serum metabolites [42], suggesting that under nutrient challenge, some level of circadian misalignment occurs among tissues. Similarly, loss of BMAL1 in the adipose tissue results in the inability of the hypothalamus to sense normally rhythmic free fatty acids from the circulation, inducing weight gain in spite of isocaloric feeding [43]. While HF diet can induce a slight change in the free running period of mice [23, 28], only hypocaloric feeding is thought to substantially alter time keeping and photic response in the SCN [44]. Collectively, these data suggest that a HF diet induces both circadian misalignment across peripheral tissues and the SCN but also potentially misaligns peripheral tissues with hypothalamic nuclei involved in nutrient sensing and energy intake. Our data provides evidence that the insulin sensitivity of a tissue may have something to do with this misalignment of clocks.

While this study reveals that treatment of an obese, insulin-resistant rodent with the antidiabetic drug, ROSI, reverses aberrant BMAL1 recruitment in spite of continued HF feeding, subsequent studies analyzing the effects of alternative insulin sensitizing agents should be performed. In addition, it will be helpful to know whether tissue-specific models of insulin receptor deficiency have altered BMAL1 recruitment and activity independent of HF diet feeding. Such studies will be necessary to fully confirm whether HF diet-induced reprogramming is secondary to the development of insulin resistance.

It is interesting that while ROSI restores BMAL1 recruitment in a target-specific manner in the tissues examined, prior studies reveal that while ROSI improves hepatic sensitivity, it neither reduces hepatic lipid deposition nor obesity in humans and rodents [45, 46]. Thus, the normalizing effect on the clock observed in mice (which may also be occurring in patients taking thiazolidinediones) may be due to some interplay between BMAL1 and PPAR proteins at the level of chromatin function. While further chromatin approaches will be necessary to reveal this, our study suggests that one of the beneficial metabolic aspects of ROSI treatment may be the realignment of misaligned circadian clocks.

## Electronic supplementary material

Supplemental Material
