# Supplementary material for: Rosiglitazone reverses high fat diet-induced changes in BMAL1 function in muscle, fat, and liver tissue in mice
Source: Int J Obes (Lond). 2018 May 24;43(3):567–80. doi: 10.1038/s41366-018-0090-5 (PMC6351224; doi:10.1038/s41366-018-0090-5)
Supplement: Supplementary file 1 — Supplemental Material [file 41366_2018_90_MOESM1_ESM.docx]

Supplemental data

SF1.


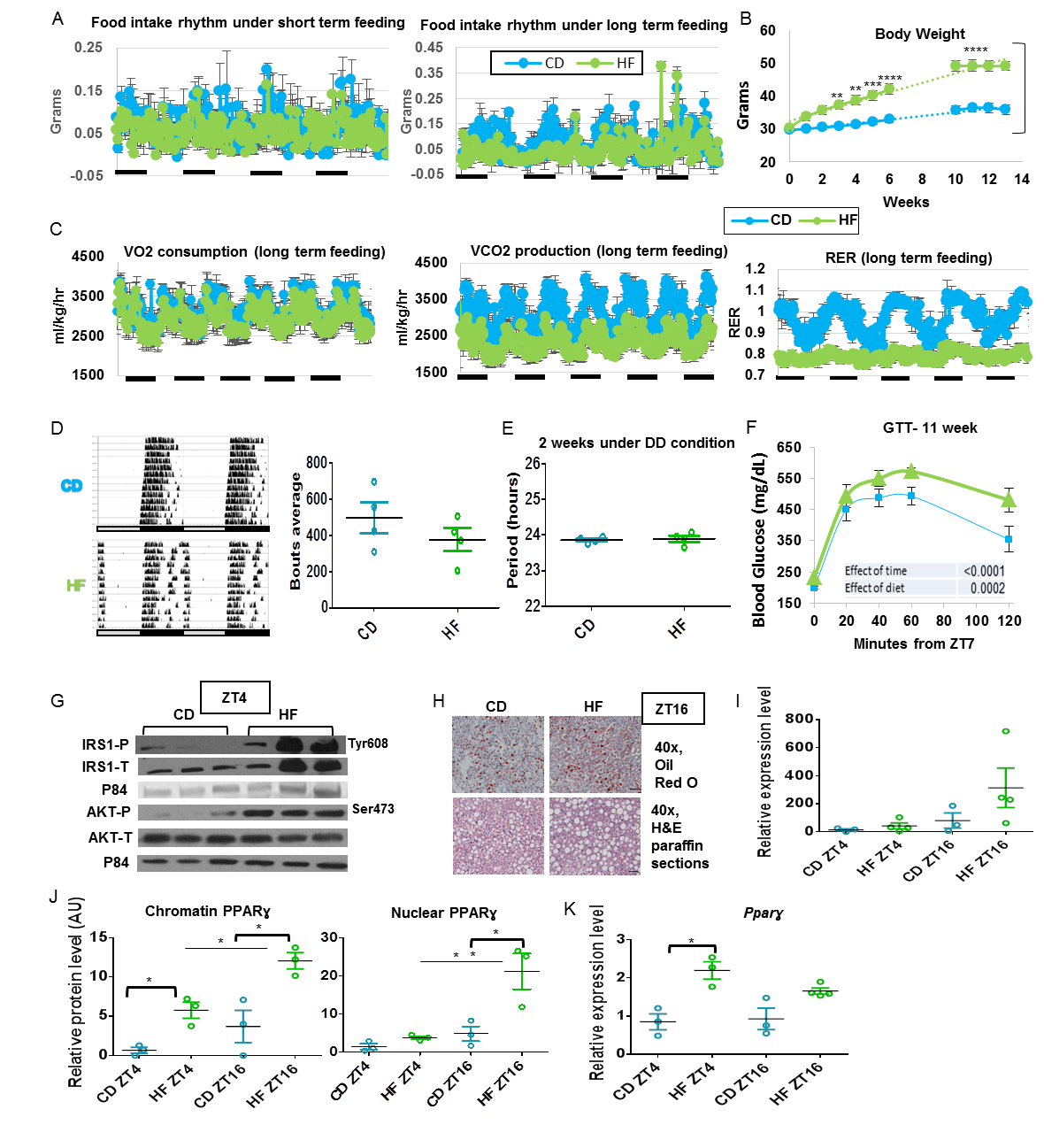


SF2.


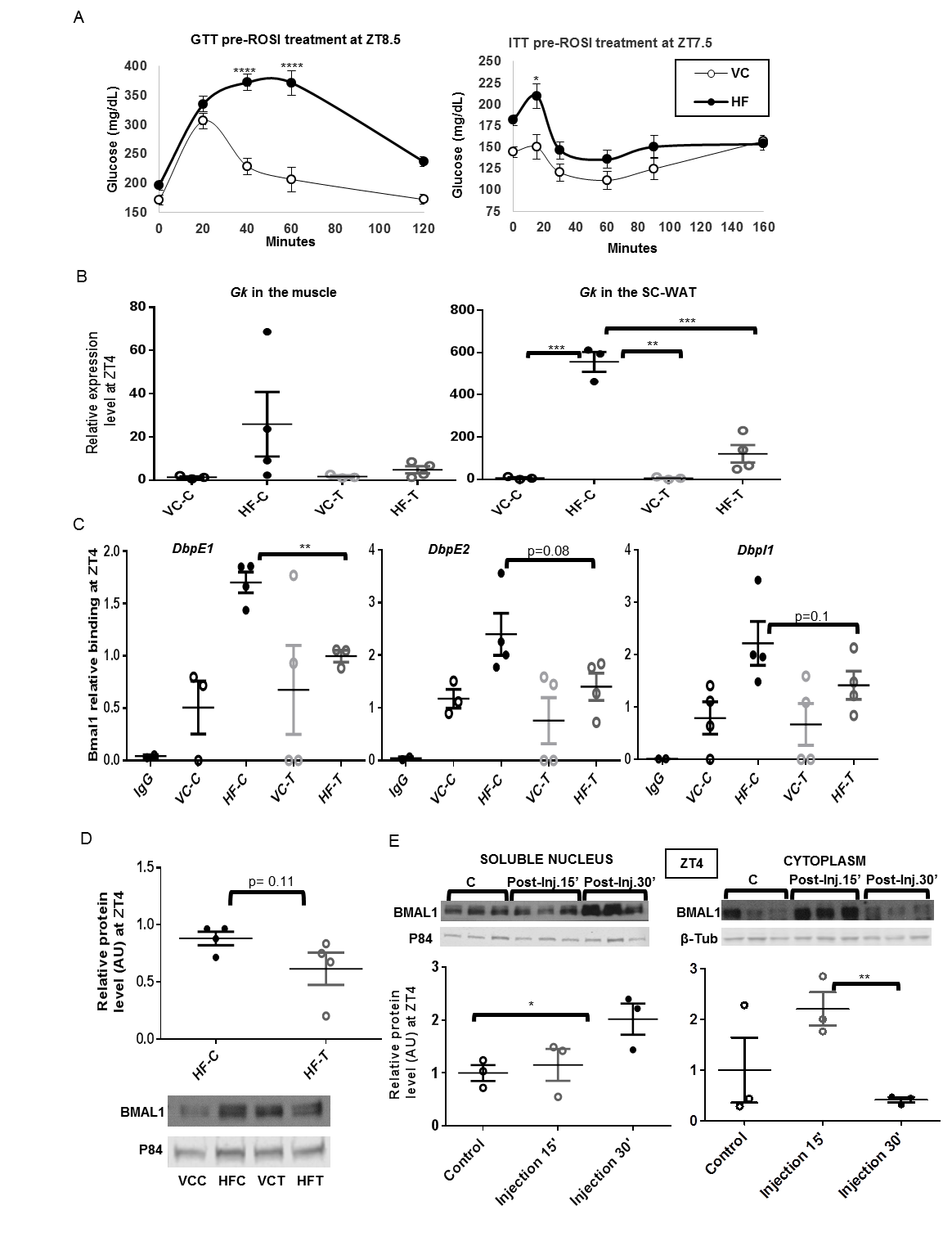


**Figure S1 Alterations in Circadian Metabolism in Animals on Vivarium Chow vs. CD and HF**

(A) Diurnal variation in food intake after transient (left panel) and chronic CD and HF feeding (right panel) (n=4). (B) Weekly body weight in grams of animals fed control chow (CD) or high fat diet (HF) for 13 consecutive weeks (n = 8). (Weight was not measured during constant dark conditions [weeks 7-9]). Significance (p < 0.05) was determined by two-way ANOVA followed by Tukey’s post hoc test. (C) Oscillations in VO2 consumption and VCO2 production and respiratory exchange ratio (RER) of animals under chronic exposure to CD and HF (n=4). (D) Wheel running of CD and HF mice under free running conditions (left panel) and wheel rotation quantification (right panel) (n = 4). (E) Free running period length of mice on CD and HF quantified in hours (n = 4). Significance (p < 0.05) was determined by Mann-Whitney *U* test (panels D-E). (F) Blood glucose levels in mice subjected to IP glucose tolerance tests (GTT) at ZT7 after eleven weeks under HF exposure (n = 8). Significance (p < 0.05) was determined by two-way ANOVA followed by Tukey’s post hoc test. (G) Western blot of whole cell lysates reveals levels of P-IRS (Tyr608), total IRS, phosphorylated AKT (ser473), total AKT and protein p84 (n=3). (H) Oil Red O and H&E staining reveals hepatic lipid deposition in animals on CD or HF diet for 13 weeks. Livers were harvested at ZT16. (I) qPCR reveals diurnal mRNA abundance of hepatic *Cidec* in CD (n=3) and HF (n=4) conditions. *Cidec* mRNA levels at ZT4 in CD were set to 1. (J) Western blot quantification reveals diurnal expression of PPARγ in the chromatin and soluble nuclear compartments of CD and HF animals (n = 3). PPARγ protein levels in CD at ZT4 were set to 1. (K) qPCR reveals diurnal mRNA abundance of hepatic *Pparγ* in CD (n=3) and HF (n=4) conditions. *Pparγ* mRNA levels at ZT4 in CD were set to 1. Significance (p < 0.05) was determined by two-way ANOVA followed by Tukey’s post hoc test (panels I-K). (*p < 0.05, **p < 0.01, ***p < 0.001, ****p < 0.0001).

**Figure S2: Rosiglitazone Restores BMAL1 Recruitment at E box Targets**

(A) Circulating glucose during glucose and insulin tolerance tests in animals fed chow (VC) or HF (n = 8). (B) qPCR analysis reveals relative mRNA abundance of *glucokinase* (*Gk*) in the muscle and subcutaneous white adipose tissue (SC-WAT) of animals treated with ROSI (T) compared to vehicle (C) (n = 4). *Gk* mRNA levels for VC-C were set to 1. Significance (p < 0.05) was determined by two-way ANOVA followed by Tukey’s post hoc test (Panels A-B) (C) Chromatin immunoprecipitation of BMAL1 from the liver reveals BMAL1 localization to target E boxes throughout the *Dbp* gene in VC and HF fed mice treated with vehicle (C) or ROSI (T) (n = 4). Significance (p < 0.05) was determined by an unpaired Student’s *t*-test comparing HF-C to HF-T. (D) Western blotting reveals nuclear BMAL1 protein in animals fed HF or VC after vehicle (C) or ROSI (T) treatment (bottom panel). Protein quantification in arbitrary units (AU) in the top panel (n = 4). Significance (p < 0.05) was determined by Mann-Whitney *U* test comparing HF-C vs HF-T. Relative BMAL1 protein level was set to 1 for HF-C. (E) BMAL1 levels in liver soluble nuclear (left panel) and cytoplasmic (right panel) fraction of animals (n = 3) fasted for 4 hours or subjected to an acute insulin injection (0.75U/kg) at ZT4 (top) and quantification in arbitrary units (AU) (bottom panel). Significance (p < 0.05) was determined by an unpaired Student’s *t* test. BMAL1 levels for the control fasted animals were set to 1. (*p < 0.05, **p < 0.01, ***p < 0.001, ****p < 0.0001).

**Supplemental Experimental Procedures**

**RNA Extraction and Reverse Transcriptase, Quantitative PCR**

Total RNA from cells and tissues was isolated with TRIzol reagent (Fisher Scientific, 15-596-018, MA, US) as described by the manufacturer. RNA was quantified by spectrophotometry (Nanodrop 2000 Spectrophotometer, Thermo Scientific, IL, US). Complementary DNA was generated using the High-Capacity complementary DNA Reverse Transcription Kit from Bio-Rad (170-8891, CA, US). A total of 10 ng of cDNA was subjected to quantitative PCR amplification using SsoAdvanced Universal SYBR Green Supermix Mix from Bio-Rad (172-5274, CA, US) on a Light Cycler instrument (C1000 Touch, Bio-Rad, CA, US). The thermal profile settings were: 95 °C for 30 sec, 95 °C for 10 sec, 62 °C for 30 sec and then 39 cycles at 65 °C for 31 sec and 65 °C for 5 sec. The eukaryotic ribosomal subunit 18S was used as an internal control. The sequence of the primers used are shown in table 1. Data were converted and normalized to the linear form by the 2^--^CT (∆∆CT) calculation (Livak, K. J. & Schmittgen, T. D. Analysis of relative gene expression data using real-time quantitative PCR and the 2(-Delta C(T)) Method. Methods 25,402–8 (2001).

**Western Blotting**

Nitrocellulose membranes probed with primary antibodies (see Materials and Methods) were washed with TTBS, and blots were incubated at a 1:15,000 dilution at room temperature for 1h with either IR Dye 800CW donkey anti-rabbit (Li-Cor, 926-32213, NE, USA), anti-mouse (Li-Cor, 926-32210, NE, USA) secondary antibodies, (H=L)-HRP conjugate anti-rabbit (Bio-Rad, 64026773, CA, USA), or anti-mouse (Bio-Rad, 64053231, CA, USA) secondary antibodies, depending on the primary antibody used (shown in table 2). The blots were then washed thoroughly in TTBS. Immunoreactive proteins conjugated with HRP were visualized with an enhanced chemiluminescence substrate kit (ECL plus; Bio-Rad, 102030812, CA, US) according to the manufacturer’s instructions. Images were obtained via developer and film for HRP conjugated proteins, or with a CLx imaging system (Odyssey classic; Li-Cor, 004-1354, NE, USA) for fluorescence proteins. Band quantification was performed with ImageJ software (NIH, USA). Results are expressed as relative intensity.

Table 1. Antibodies

| Primary antibodies | Secondary antibodies |
| --- | --- |
| BMAL1 (Abcam, ab93806, CA, US) | (H=L)-HRP conjugate monoclonal anti-rabbit |
| PPARG (Santa Cruz, Sc-7196, CA, US) | (H=L)-HRP conjugate monoclonal anti-rabbit |
| IRS1 (BD Biosciences, 611394, NJ, US) | (H=L)-HRP conjugate monoclonal anti-mouse |
| P-IRS1 (Millipore, 09-432, MA, US) | (H=L)-HRP conjugate monoclonal anti-rabbit |
| AKT (Cell Signalling, 4691S, MA, US) | IR Dye 800CW donkey anti-rabbit |
| P-AKT (Cell Signalling, 3787S, MA, US) | IR Dye 800CW donkey anti-rabbit |
| TUBULIN (Abcam, ab6046, CA, US) | IR Dye 800CW donkey anti-rabbit |
| H3 (Genetex, 122148, CA, US) | (H=L)-HRP conjugate monoclonal anti-rabbit |
| P84 (Genetex, 70220, CA, US) | IR Dye 800CW donkey anti-mouse |
| ACTIN(Abcam, ab119716, CA, US) | IR Dye 800CW donkey anti-mouse |

Table 2. Protein fractionation buffers

| **Protein fraction** | **Buffer used** | **Buffer recipe** |
| --- | --- | --- |
| Whole Cell Isolates and Chromatin | RIPA | 50 mM Tris, 150 mM NaCl, EDTA 5mM, MgCl_2_ 15mM and NP-40 1% |
| Cytoplasm | Buffer A | 100 mM HEPES pH 7.8, 250 mM KCL, 1.5 mM spermine, 5mM spermidine, 10 mM EGTA, 10 mM EDTA, 3.2 M Sucrose, cold 3% triton) |
| Soluble Nucleus | Low/High Salt Buffer | Low Salt Buffer: 100 mM HEPES pH 7.8, 250 mM KCL, 1.5 mM spermine, 5mM spermidine, 10 mM EGTA, 10 mM EDTA, 20% glycerol.  High Salt Buffer: Same + 0.5 M KCl |

*Freshly added protease inhibitor tablet 50x (Roche), 1M NaF, 400mM NAM, 3.3 mM TSA and 100 mM PMSF (Sigma-Aldrich) were included for each buffer.

Table 3. Primers

| **NAME** | **SEQUENCE** |
| --- | --- |
| Bmal1 FW | GCAGTGCCACTGACTACCAAGA |
| Bmal1 RV | TCCTGGACATTGCATTGCAT |
| Cidec FW | CAGAAGCCAACTAAGAAGATCG |
| Cidec RV | TGTAGCAGTGCAGGTCATAG |
| Chrono FW | ACTCAAGATGGGTCGCTTTG |
| Chrono RV | GGGCAGCTATGTGAGGAAAC |
| Chrono chiP FW | CACGGCTGGAGTGTACAGAG |
| Chrono chiP RV | GAAAGAGTGGGGAGTCACGA |
| Dbp FW | AATGACCTTTGAACCTGATCCCGCT |
| Dbp RV | GCTCCAGTACTTCTCATCCTTCTGT |
| DbpE1_FW | TCTGCAGAACTGACTGGTTGA |
| DbpE1_RV | GCGTGCAAGCCTCCAGGAT |
| DbpE2 FW | ACCGTGGAGGTGCTAATGAC |
| DbpE2 RV | CTCCTCTGAGAAGCGGTGTC |
| DbpUp_FW | ACACCCGCATCCGATAGC |
| DbpUp_RV | CCACTTCGGGCCAATGAG |
| Dbpi1 Fw | ATGCTCACACGGTGCAGACA |
| Dbpi1 Rv | CTGCTCAGGCACATTCCTCAT |
| Fas FW | ATCCCAGCACTTCTTGATGG |
| Fas RV | CCGAAGCCAAATGAGTTGAT |
| Fabp2 FW | GTGGAAAGTAGACCGGAACGA |
| Fabp2 RV | CCATCCTGTGTGATTGTCAGTT |
| Fabp2 chiP FW | CGGCACGTGGAAAGTAGACC |
| Fabp2 chiP RV | AGGAAGCCAGTGCTTACCCA |
| Gk FW | GATCCGGGAAGAGAAGCAAG |
| Gk RV | GACAGGGATGAGGGACAGAG |
| PcxF | GATGACCTCACAGCCAAGCA |
| PcxR | GGGTACCTCTGTGTCCAAAGGA |
| PparG FW | CAAGAATACCAAAGTGCGATCAA |
| PparG RV | GAGCTGGGTCTTTTCAGAATAATAAG |
| Per2 FW | CGCCTAGAATCCCTCCTGAGA |
| Per2 RV | CCACCGGCCTGTAGGATCT |
| 18s FW | CGCCGCTAGAGGTGAAATTC |
| 18S RV | CGAACCTCCGACTTTCGTTCT |
